# Association between anticholinergic burden and volumes of the basal forebrain and hippocampus across the Alzheimer’s disease spectrum ‐ data from the DELCODE study

**DOI:** 10.1002/alz.087239

**Published:** 2025-01-09

**Authors:** Ingo Kilimann, Jonas Peltner, Fedor Levin, Annika Spottke, Oliver Peters, Emrah Düzel, Frank Jessen, Britta Haenisch, Stefan Teipel

**Affiliations:** ^1^ Department of Psychosomatic Medicine, Rostock University Medical Center, Rostock Germany; ^2^ Deutsches Zentrum für Neurodegenerative Erkrankungen e. V. (DZNE) Rostock/Greifswald, Rostock Germany; ^3^ Deutsches Zentrum für Neurodegenerative Erkrankungen e. V. (DZNE) Bonn, Bonn Germany; ^4^ Department of Neurology, University of Bonn, Bonn Germany; ^5^ Deutsches Zentrum für Neurodegenerative Erkrankungen e. V. (DZNE) Berlin, Berlin Germany; ^6^ Charité – Universitätsmedizin Berlin, corporate member of Freie Universität Berlin and Humboldt‐Universität zu Berlin – Institute of Psychiatry and Psychotherapy, Berlin Germany; ^7^ German Center for Neurodegenerative Diseases (DZNE), Magdeburg Germany; ^8^ Institute of Cognitive Neurology and Dementia Research (IKND), Otto‐von‐Guericke University, Magdeburg Germany; ^9^ Department of Psychiatry and Psychotherapy, Medical Faculty, University of Cologne, Cologne Germany; ^10^ Research Division, Federal Institute for Drugs and Medical Devices (BfArM), Bonn Germany

## Abstract

**Background:**

Drugs with desirable and undesirable anticholinergic effects influence cognitive performance and increase the risk of dementia later in life. MRI imaging studies showed structural effect of these substances on basal forebrain and hippocampus, key regions of Alzheimer’s disease pathology.

**Methods:**

We analyzed data from 787 participants of the DELCODE study with a diagnosis of subjective cognitive impairment (SCD), mild cognitive impairment, Alzheimer’s disease dementia, 1st degree relatives of people with dementia and cognitively healthy controls. We tested for associations between basal forebrain and hippocampal volume and anticholinergic medication exposure using the anticholinergic burden (ACB) score, modified for drugs approved in Germany. Furthermore, we investigated the influence of ApoE 4 status on possible effects.

**Results:**

Among all cases, we found no statistically significant association between the ACB sum score and volume of hippocampus or basal forebrain. Within diagnostic groups, an association between ACB score and hippocampus volumes was only detectable in participants with a diagnosis of SCD; all other diagnostic groups showed no association. Dividing the SCD group into ApoE 4 positive and negative status, the significant effect on hippocampus volume only remained in the ApoE 4 negative group. Additionally, there was a significant effect on the basal forebrain volume. No associations were seen in the ApoE 4 positive group.

**Conclusion:**

A structural effect of anticholinergic load was shown in this cohort as in previous studies. It is interesting to note that the association can only be mapped in the group of people with a subjective cognitive disorder without an ApoE 4 risk allele. An effect of the anticholinergic burden could therefore be particularly relevant if no other risk factors such as ApoE 4 are present.

